# Synthesis of Poly(1,4‐anthraquinone) Using Catalytic Amounts of Nickel

**DOI:** 10.1002/advs.202506251

**Published:** 2025-07-21

**Authors:** Florin Adler, Sebastián Pinto‐Bautista, Christoph Lorenz, Lars Hinrichs, Marcel Weil, Birgit Esser

**Affiliations:** ^1^ Institute of Organic Chemistry II and Advanced Materials Ulm University Albert‐Einstein‐Allee 11 89081 Ulm Germany; ^2^ Helmholtz Institute Ulm (HIU) Helmholtzstraße 11 89081 Ulm Germany; ^3^ Institute for Technology Assessment and System Analysis (ITAS) Karlsruhe Institute of Technology (KIT) Karlstraße 11 76133 Karlsruhe Germany

**Keywords:** catalysis, multivalent batteries, P14AQ, redox polymers, Yamamoto polymerization

## Abstract

Multivalent metal batteries are attractive options to diversify energy storage options offering higher sustainability, but the high charge density of multivalent ions poses challenges to electrode materials. Organic electrode materials are currently the best‐performing candidates, and among them, poly(1,4‐anthraquinone) (**P14AQ**) shows excellent electrochemical properties. This holds in particular for magnesium‐ion batteries, where the polymer displays reversible insertion of Mg^2+^ ions with high cycling stability. The conventional synthesis of **P14AQ**, however, employs stoichiometric amounts of bis(cycloocta‐1,5‐diene)nickel(0) (Ni(COD)_2_), which is an air‐ and light‐sensitive and expensive chemical, and therefore lacks sustainability. Herein a synthetic route to **P14AQ** is presented that uses only catalytic amounts of dibromobis(triphenylphosphine)nickel(II) (NiBr_2_(PPh_3_)_2_). This route – in addition to being more cost‐efficient and less toxic – results in higher yields and polymer molecular weights. A life‐cycle assessment (LCA) comparing the new and conventional polymerization methods shows that regarding the environmental impact categories climate change, human toxicity, and cumulative energy demand, the new method brings significant improvement.

## Graphical abstract

**P14AQ** is an excellent battery electrode material, especially for multivalent battery cells. A new synthetic route is presented using stable and easy‐to‐handle dibromobis(triphenylphosphine)nickel(II) (NiBr_2_(PPh_3_)_2_) in catalytic amounts instead of stoichiometric amounts of air‐ and temperature‐sensitive bis(cyclooct‐1,5‐diene)nickel(0) (Ni(COD)_2_). The new route furnishes **P14AQ** in higher yields and average molecular masses and improves the environmental impact categories of climate change, human toxicity, and cumulative energy demand.

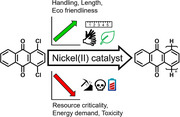

## 1 Introduction

Lithium‐ion batteries are widely used in electronic devices, ranging from mobile to stationary storage. Due to the relatively low earth abundance of lithium compared to other alkali and alkaline earth metals, it is of great interest to focus research on the latter‐type batteries, so‐called post‐lithium storage options.^[^ ^1^, ^2^ ^]^ However, in particular in multivalent metal‐based batteries, the search for compatible positive electrode materials regarding output voltage, cyclability, (electrochemical) stability, and reversibility of charge and discharge processes faces challenges.^[^ ^3^, ^4^ ^]^ This is in particular due to the high charge density of multivalent metal cations, leading to sluggish ion migration in inorganic transition metal oxides. Here, organic electrode materials are promising candidates, characterized by weaker interactions of the metal ions with the organic redox‐active sites and more amorphous morphologies.^[^ ^5^, ^6^ ^]^ For a reversible reduction during discharge of the battery, associated with a reversible uptake of multivalent metal ions, *para*‐quinoid structures seem particularly well suited.^[^ ^7^, ^8^ ^]^ They belong to the category of n‐type materials, with anthraquinone being the most‐studied example. To counteract its dissolution into liquid electrolytes, anthraquinone is typically embedded into a polymeric architecture.^[^ ^9^ ^]^

Polyanthraquinones, and especially poly(1,4‐anthraquinone) (**P14AQ**), belong to the most promising quinone‐based polymers as electrode materials, in particular for multivalent metal batteries.^[^ ^10^ ^]^ Upon its first report, **P14AQ** was investigated in organic lithium‐based batteries with remarkable capacity retention and cycling performance.^[^ ^11^ ^]^ **P14AQ** shows not only excellent behavior in lithium batteries, but also in lithium‐magnesium hybrid^[^ ^12^ ^]^ and magnesium metal^[^ ^13^, ^14^, ^15^ ^]^ batteries. It is also an excellent candidate for aqueous potassium batteries,^[^ ^16^, ^17^ ^]^ polymer‐air^[^ ^16^ ^]^ or zinc‐based batteries.^[^ ^18^ ^]^ In magnesium batteries, a particular advancement was achieved with the use of a chloride‐free electrolyte, namely the Mg[B(hfip)_4_]_2_ salt with the weakly coordinating ion^[^ ^19^ ^]^ (hfip = OC(H)(CF_3_)_2_)) in glymes,^[^ ^20^ ^]^ which enables reversible insertion of Mg^2+^ cations,^[^ ^21^ ^]^ together with a **P14AQ**‐based positive electrode. To improve the polymer's performance even further, attempts using a **P14AQ/**CNTs (carbon nanotube)‐hybrid material were made and tested versus lithium.^[^ ^22^ ^]^ **P14AQ** has not only been used as a battery electrode material, but also as an anchor for carbon capture.^[^ ^23^ ^]^

**Scheme 1:**
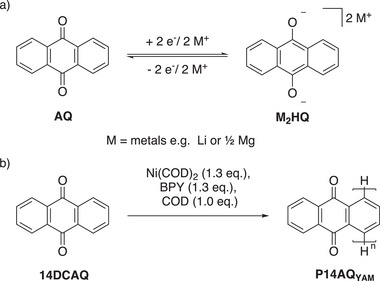
a) Electrochemical redox process of the monomer unit AQ and its reduced form M_2_HQ; b) Conventional synthesis of P14AQ_yam_ using a yamamoto polymerization of 14DCAQ.

The basis for its application as a battery electrode material is that the central *p*‐quinone unit in anthraquinone (**AQ**) can accept two electrons in a reversible redox process forming the dimetallated hydroquinone **M_2_HQ** (M = Li or ½ Mg, **Scheme** **1**). Until today the polymer **P14AQ** is synthesized via the yamamoto polymerization^[^ ^12^ ^]^ of 1,4‐dichloroanthracene‐9,10‐dione (**14DCAQ**) using a stoichiometric amount of bis(cycloocta‐1,5‐diene)nickel (Ni(COD)_2_), which is an air‐ and light‐sensitive as well as rather expensive chemical (Scheme 1).^[^ ^24^ ^]^ Due to its large surface area, Ni(COD)_2_ has a higher reactivity toward oxidation, such as through oxygen, compared to pure metal. To prevent decomposition over time, it should be stored in an oxygen‐ and light‐free environment. To decrease the overall usage of nickel and simplify the storage of employed chemicals, this work focuses on a modification of the synthesis of **P14AQ** from **14DCAQ** using only a catalytic amount of Ni(II) salt as air‐ and temperature‐stable compounds.

In the conventional yamamoto polymerization using Ni(COD)_2_, nickel changes its oxidation state from 0 to +II throughout the reaction and by consumption of the reagent (see Scheme S2, Supporting Information). In order to turn the reaction into one that uses only a catalytic amount of nickel, a reducing agent can be added, which reduces the formed Ni(II) back to Ni(0) in order to undergo another cross‐coupling cycle (see Scheme S3, Supporting Information). The nickel reagent can even be employed in the +II oxidation state and be reduced to Ni(0) before the very first cross‐coupling cycle. For a successful use of such a catalyst system, the reducing agent must be able to change the oxidation state of the catalyst (from Ni(II) to Ni(0)) and at the same time not interfere with or change the reaction itself. Already in 1990 ichikawa and coworkers published a method using metallic zinc as a reducing agent and NiCl_2_ as a precatalyst for a Ni‐catalyzed polymerization of aromatic dichlorides forming aromatic poly(ether ketone)s.^[^ ^25^ ^]^ Surprisingly, in the past ten years since **P14AQ** has been discovered no nickel‐catalyzed polymerization of **14DCAQ** has been developed.

We herein report a Ni‐catalyzed polymerization method for the synthesis of **P14AQ** from **14DCAQ** using dibromobis(triphenylphosphine)nickel(II) (NiBr_2_(PPh_3_)_2_) as precatalyst together with stoichiometric amounts of zinc. We found that this method provides the **P14AQ** polymer with molar masses even exceeding those obtained with the conventional yamamoto polymerization. We performed a detailed life‐cycle assessment (LCA) comparing the new polymerization method to the conventional yamamoto route regarding a range of aspects. This showed that in the areas of climate change, human toxicity, and cumulative energy demand, the new method brings significant improvement while providing guidance for further development of the reaction to improve all environmental aspects.

## 2 Results and Discussion

For simplicity, from here on **P14AQ** synthesized by the conventional yamamoto polymerization will be labeled **P14AQ _yam_ **, and that synthesized by our new Ni‐catalyzed method **P14AQ_cat_**.

### 2.1 Development of the Synthesis of **P14AQ_cat_**

To substitute the stoichiometric use of Ni(COD)_2_ we were inspired by reports from ichikawa and coworkers from 1990,^[^ ^25^ ^]^ who used NiCl_2_ as a catalyst and Zn as a reducing agent for the Ni(II) salts in a polymerization of aromatic dichlorides to poly(ether ketone)s. For the polymerization of **14DCAQ** we therefore tested different commercially available Ni(II) sources as the catalyst (see **Table** **1**), added 2,2′‐bipyridine (BPY) as a ligand, and triphenylphosphine (PPh_3_) to suppress unwanted side reactions.^[^ ^25^ ^]^ The use of an iodide source to increase the yield of nickel‐catalyzed couplings has been known for almost 60 years,^[^ ^26^ ^]^ and, therefore, we added 1.0 eq. of potassium iodide (KI) to the polymerization reactions. To evaluate the effectiveness of the polymerization, we used a Matrix‐Assisted Laser‐Desorption/Ionization (MALDI)‐Time‐of‐Flight (ToF) spectrometric analysis of the product samples and compared the results to those of the conventional polymerization using the stoichiometric yamamoto conditions^[^ ^11^ ^]^ (**P14AQ _yam_ **). Under the standard yamamoto reaction conditions (see Scheme 1), **P14AQ _yam_** is obtained with a number‐averaged molar mass (*M̅*_n_) of 4880 g mol^−1^, a mass‐averaged molar mass (*M̅*_w_) of 5440 g mol^−1^, a dispersity (*Đ*) of 1.12 and a degree of polymerization (*DP*) of 24 (see **Table** **2**, Entry 1).

**Table 1:** Overview of the catalyst screening for the Ni‐catalyzed polymerization of **14DCAQ** to **P14AQ_cat_**.

Entry^a)^	Catalyst	Amount [mol%]	*M̅*_n_ [g mol^−1^]	*M̅*_w_[g mol^−1^]	*Đ*	*DP*
1	NiCl_2_	10	3590	4460	1.23	18
2	NiBr_2_	10	2120	2780	1.31	10
3	Ni(OAc)_2_	10	3650	4830	1.32	18
4	NiBr_2_(PPh_3_)_2_	10	3490	4430	1.27	17
5	NiBr_2_(PPh_3_)_2_	20	6340	6790	1.07	31
6	NiCl_2_(PPh_3_)_2_	20	6200	6740	1.09	30
7	NiCl_2_(dppe)	20	2830	3940	1.39	14
8	NiCl_2_(dppp)	20	2590	3220	1.25	13
9	NiCl_2_(dppf)	20	–	–	–	–
10	NiCl_2_	20	2570	2930	1.14	13
11	NiBr_2_	20	4190	4840	1.12	21
12	Ni(OAc)_2_	20	2220	2390	1.08	11

^a)^ All screening attempts were performed on a 0.3 mmol scale. The number‐averaged molar mass (M̅_n_), mass‐averaged molar mass (M̅_w_), dispersity (Đ) and the degree of polymerization (DP) were calculated using the bruker MS software polytool.

**Table 2:** Scale up of optimized reaction conditions for the Ni‐catalyzed polymerization of **14DCAQ** to **P14AQ_cat_** (Scheme 3) in comparison with the conventional yamamoto conditions **P14AQ_yam_** (Scheme 1).

Entry^a)^	Method	*M̅*_n_ [g mol^−1^]	*M̅_w_* [g mol^−1^]	*Đ*	*DP*
1	yamamoto ^b)^	4880	5440	1.12	24
2	Ni catalyzed (0.6 m)^c)^	5790	6330	1.09	28

^a)^ The polymerizations were performed on a 2.1 mmol scale. The number‐averaged molar mass (M̅_n_), mass‐averaged molar mass (M̅_w_), dispersity (Đ), and the degree of polymerization (DP) were calculated using the bruker MS software polytool;

^b)^ Reaction conditions of the standard yamamoto polymerization: **14DCAQ**, Ni(COD)_2_ (1.3 eq.), BPY (1.3 eq.), COD (1.0 eq.), DMF (0.6 m), 60 °C, 69 h;

^c)^ Reaction conditions of the Ni‐catalyzed polymerization: **14DCAQ**, NiBr_2_(PPh_3_)_2_ (20 ml%), BPY (20 mol%), Zn (3.0 eq.) and KI (1.0 eq.), DMF, 60 °C, 69 h. (COD = 1,5‐cyclooctadiene).

**Scheme 2:**
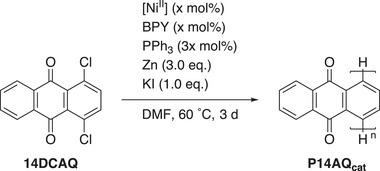
Reaction conditions of the Ni‐catalyzed polymerization of **14DCAQ** to **P14AQ_cat_** with a Nickel(II) salt ([NiII]).

Since the new catalyst systems tested consisted of the Ni(II) salt, 2,2′‐bipyridine (BPY) in equimolar amounts, and PPh_3_ in triple‐molar amounts, an increase in salt accordingly resulted in an increase in the used amounts of bipyridine and phosphine (**Scheme** **2**, Table 1). The first attempt at the polymerization using NiCl_2_ (10 mol%) as a catalyst only furnished oligomers with number‐averaged molecular weights of *M̅*_n_  =  3590 g mol^−1^ (Table 1, Entry 1). We then tested different catalysts, but observed no improvement regarding the polymer length with 10 mol% each of NiBr_2_ or Ni(OAc)_2_ (Table 1, Entries 2 and 3). The reaction with 10 mol% NiBr_2_(PPh_3_)_2_ also did not furnish higher molecular weights, but produced an insoluble black residue, which had not been observed in previous attempts (Entry 4). This residue probably resulted from the catalyst reaching the limits of its turn‐over number,^[^ ^27^ ^]^ in which case an increase of catalyst should improve the polymerization. Therefore, we next increased the catalyst loading to 20 mol% NiBr_2_(PPh_3_)_2_ (Entry 5). Indeed, here the polymers reached significantly higher molecular weights of over 6000 (*M̅*_n_  =  6340 g mol^−1^). Similar results were achieved when using NiCl_2_(PPh_3_)_2_ (Entry 6), but not with any of the catalyst employed in the following. With NiCl_2_(dppe) and NiCl_2_(dppp), polymer molecular weights below 3000 g mol^−1^ (*M̅*_n_  =  2830 g mol^−1^ and *M̅*_n_  =  2590 g mol^−1^) were obtained, and with NiCl_2_(dppf), no polymer was formed at all (Entries 7–9). Increasing the used quantity of the previously mentioned NiCl_2_, NiBr_2,_ and Ni(OAc)_2_ to 20 mol% only slightly improved the polymerization with NiBr_2_ (Entry 11), but visibly worsened it with NiCl_2_ and Ni(OAc)_2_ (Entries 10 and 12).

Since the reaction with NiBr_2_(PPh_3_)_2_ (20 mol%)) showed the most promising result, subsequent reactions were only executed with said catalyst. An additional increase in catalyst loading (25, 30, 35, 40, 60, 80, or 100 mol%, see Table S1, Supporting Information) did not further improve the polymerization, but instead led to an increase in impurities, indicated by the presence of residues insoluble in CH_2_Cl_2_ during the preparation of the mass spectrometry samples and broader signals while analyzed with MALDI‐ToF spectrometry (see Table S1, Supporting Information).

Subsequently, the importance of BPY as a ligand was tested. We investigated the use of its derivatives 4,4′‐dimethyl‐2,2′‐bipyridine, phenanthroline, or 4,7‐diphenyl‐1,10‐phenanthroline in comparison to BPY. However, a change in ligand did not further improve the polymerization. While with 4,4′‐dimethyl‐2,2′‐bipyridine we achieved the same results as with BPY, with phenanthroline and 4,7‐diphenyl‐1,10‐phenanthroline we observed a decrease in reactivity, resulting in shorter polymer chain length (see Table S2, Supporting Information).

Next, we stepwise investigated the used amount of PPh_3_ by varying it between 0–60 mol% (see Table S3, Supporting Information). Surprisingly, the polymer lengths decreased with an increasing amount of phosphine and showed the best results with no additional PPh_3_ added at all (additional, as the Ni(II) source NiBr_2_(PPh_3_)_2_ is a phosphine complex). Additionally, besides for the cases with 0 and 10 mol%, the more phosphine was added at the start of the reaction, the more impurities and shorter oligomers were present, even after purification of the product. PPh_3_ turned out not to be a necessary ingredient, and employing no extra phosphine in the reaction resulted in overall longer chain lengths and fewer impurities after workup.

**Scheme 3:**
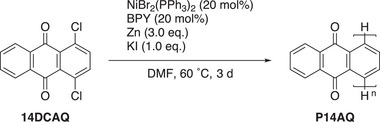
Optimized reaction conditions for the Ni‐catalyzed polymerization of **14DCAQ** to furnish **P14AQ_cat_**.

Our newly developed procedure for the synthesis of **P14AQ_cat_** using only catalytic amounts of Ni(II) is shown in **Scheme** **3**. With these improvements **P14AQ_cat_** can be now synthesized using NiBr_2_(PPh_3_)_2_ and BPY (each 20 mol%) as catalyst system, Zn as the reducing agent (3.0 eq.), and KI as additive (1.0 eq.) in *N*,*N*‐dimethylformamide (DMF) at 60 °C with a reaction time of three days (Scheme 3) in yields of 95–98% and number‐averaged molecular masses of over 6000 g mol^−1^. Finally, we tested whether this new method also works well on a larger scale (Table 2). An increase of the previously used 0.3 mmol to a 2.1 mmol scale led to an excellent number‐averaged molar mass of *M̅*_n_  =  5790 g mol^−1^, only 400 g mol^−1^ lower than on the small‐scale reaction (Table 2, Entry 2).

### 2.2 Environmental Assessment of the Synthesis of P14AQ

Conducting an environmental assessment of the newly developed synthesis route for **P14AQ_cat_** is essential to comprehensively evaluate its sustainability profile and to gain insight into how it compares to the conventional yamamoto process. While the new route may offer advantages, such as the substitution of the reactive Ni(COD)₂ and increased reaction yields, a systematic analysis is necessary to identify potential environmental hotspots and to further guide the technology development. The Life Cycle Assessment (LCA) method is a widely used approach for evaluating the potential environmental impacts of a system. This holistic and systematic method provides a clearer understanding of the broader environmental implications of a product or service, which can be used for process optimization, system comparison, burden‐shifting prevention and to support the technology development process.^[^ ^28^ ^]^ When assessing systems at early technology‐readiness levels (TRL), where the degree of uncertainty around its development is high, the focus should especially lie on so‐called environmental “hotspots”. This analysis may provide first insights to derive recommendations for further system development. In this study, an LCA of two different laboratory‐scale synthesis routes of **P14AQ**, namely the “conventional yamamoto polymerization” and the “Ni‐catalyzed polymerization”, was conducted to highlight the environmental advantages and disadvantages of each route, which were presented in Schemes 1 and 3. The LCA method follows the guidelines described in the ISO standards 14040/14044^[^ ^29^, ^30^ ^]^ and quantifies resource use, emissions, and waste generation across different stages within the life cycle of a product or service. These mass and energy flows are consequently translated into environmental impacts used to determine the environmental profile of the system. The method comprises four main steps: 1) goal and scope definition, 2) life‐cycle inventory analysis, 3) life‐cycle impact assessment, and 4) interpretation of results. Details of each step are presented in the Supporting Information, including the complete set of life cycle inventories created for this study. Four environmental impact categories of the ILCD method are displayed and discussed, while the complete set of results for all 16 categories is also included in the Supporting Information. The displayed impact categories are:

1. Acidification: indicates the potential damage to soil and waters from the release of acidifying agents, measured in molc (moles of charge) H^+^ equivalent [molc H^+^ eq];
2. Climate change: relates to the emission of CO_2_ and other greenhouse gases, measured in kg CO_2_ equivalent.
3. Mineral, fossil, and renewable resource depletion: herein abbreviated as “resource depletion,” relates to the depletion of natural resources due to human activity. It is measured in kg of Antimony (Sb) equivalent [kg Sb eq].
4. Human toxicity – carcinogenic: indicates the potential harm on human health caused by exposure to chemical substances released at each life‐cycle stage. It is expressed in Comparative Toxic Units for Humans (CTUh).

An additional calculation using the Cumulative Energy Demand (CED) method, which quantifies the total embedded energy in a system (MJ‐eq), was conducted to assess the energy intensity of each process.

**Figure 1:**
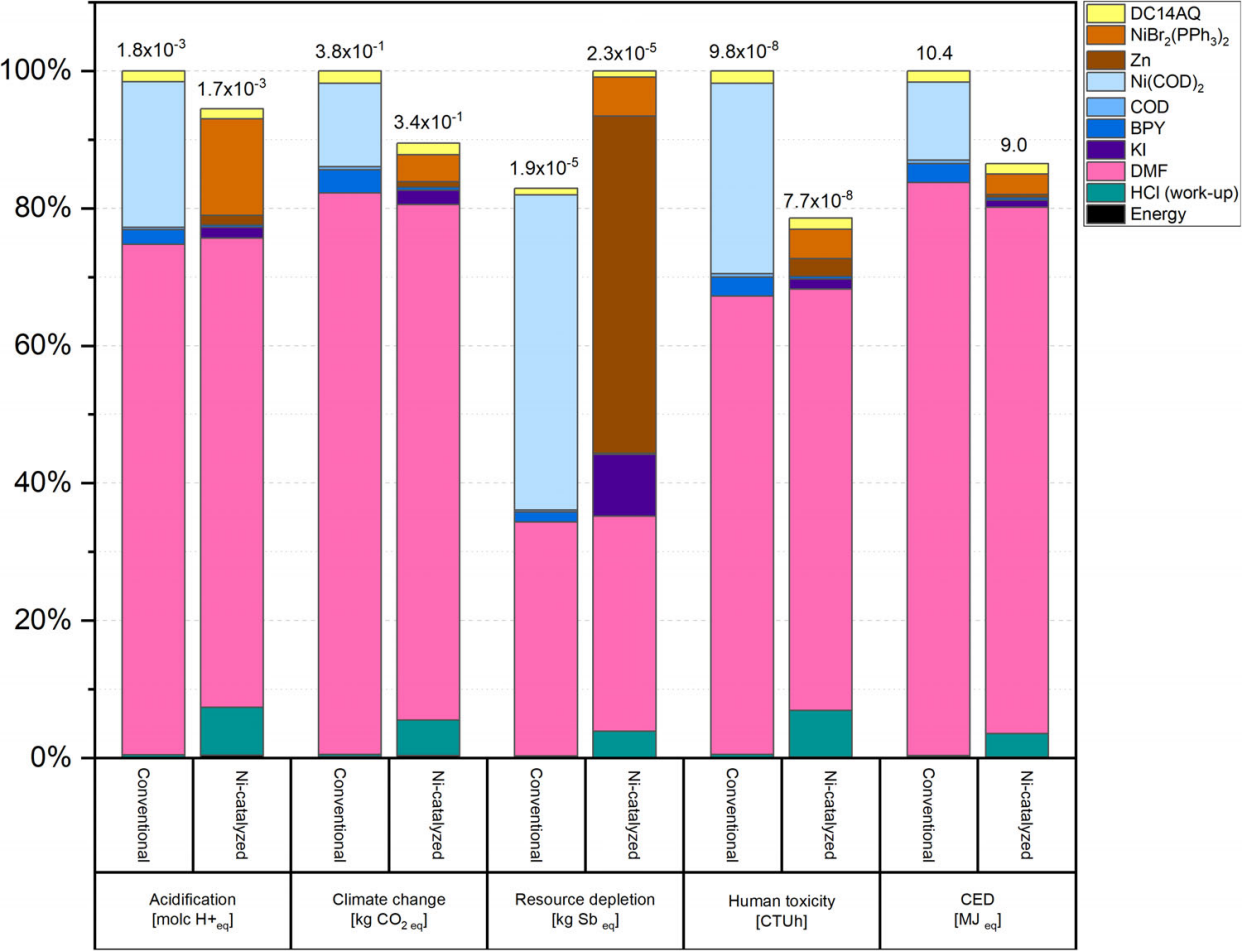
Results of the LCA for the conventional YAMAMOTO and the newly developed Ni‐catalyzed synthesis of P14AQ. (CED  =  cumulative energy demand.

A comparison of the relative LCA results for both the conventional yamamoto and Ni‐catalyzed polymerization routes of **P14AQ** (**Figure** **1**), which also account for the (aqueous) work‐up of the reactions, indicates overall comparable performance while suggesting that the Ni‐catalyzed route could achieve ≈6% lower acidification impacts, 11% lower climate change impacts and 21% lower human toxicity potential. Additionally, the Cumulative Energy Demand (CED) could be reduced to ≈86% compared to the classic yamamoto process. In contrast, the resource‐depletion potential increases ≈17% relative to the reference process. A breakdown of specific contributions reveals that the use of DMF as a solvent has the largest influence on the results, accounting for 61–83% of total impacts in acidification, climate change, human toxicity, and CED for both processes. These significant impacts primarily originate from the supply chain of carbon monoxide and dimethylamine, which are key precursors in DMF production. The use of Ni(COD)₂ as a reagent in the conventional yamamoto process also has a substantial impact on its environmental profile, ranking as the second‐highest contributor in all studied categories. For the Ni‐catalyzed route, the sourcing of nickel contained within the catalyst NiBr₂(PPh₃)₂ has considerable effects on acidification, while zinc, used as a reducing agent, is the largest contributor to resource‐depletion impacts. Specifically, the environmental burden of Zn mainly stems from ore mining, where valuable minerals may be lost due to their low concentration in ores and tailings, making their recovery economically unattractive.^[^ ^31^ ^]^ Furthermore, in the Ni‐catalyzed route, additional HCl is required for quenching of the Zn powder during work‐up, which increases the overall environmental impact of the process, particularly in terms of human toxicity. In contrast, the effects of energy demand are relatively minor, contributing less than 1% to the total impacts across all categories for both processes.

Replacing Ni(COD)₂ in the conventional yamamoto process with NiBr₂(PPh₃)₂ as a catalyst in the newly developed route introduces a trade‐off in the environmental footprint of the process. On one hand, it significantly increases the burden of resource criticality due to the additional demand for zinc; on the other hand, it reduces impacts in three of the remaining studied categories. Moreover, this substitution eliminates the need for specialized material handling and storage measures required for Ni(COD)₂, which, due to its high reactivity toward oxygen and temperature sensitivity, must be handled in inert atmospheres and at low temperatures to prevent degradation.^[^ ^32^ ^]^ Implementing such precautions would result in additional environmental burdens, which, for simplicity, have not yet been considered in this study. Additionally, in the conventional yamamoto process, Ni(COD)₂ is used as a reagent, meaning that the Ni undergoes a permanent change in oxidation state from Ni(0) to Ni(II), rendering it non‐reusable. In contrast, in the Ni‐catalyzed route, the oxidation state of NiBr_2_(PPh_3_)_2_ remains unchanged, making catalyst recovery and repurposing feasible. Similarly, the excess of the reducing agent (Zn) could be recovered by dissolving the polymer in chloroform and separating the solution from the unreacted Zn powder before recycling/reusing or quenching. Such material‐recovery practices are common in industrial processes and have the potential to further reduce the environmental impact of this synthesis route. It is recommended, for both processes, to address proper DMF recycling with high recycling rates to decrease significantly the environmental impacts.

The results of this LCA highlight hotspots of environmental burdens that could translate into areas for process optimization, while also suggesting potential advantages of the Ni‐catalyzed process over the conventional yamamoto method. These results are subject to a degree of uncertainty due to the limitations inherent in the data sources and modelling approaches used, which are normal in early TRL.^[^ ^33^ ^]^ Specifically, some life‐cycle inventories of agents and precursors have been constructed based on information derived from a variety of literature sources (including patents) and stoichiometric calculations, which, while providing valuable insights, may not fully capture the complexity of real‐world processes. Patents, for instance, often describe laboratory conditions that may differ from industrial‐scale operations, and stoichiometric calculations, though useful for estimating material flows, can overlook important factors such as reaction efficiencies, side products, and process‐specific energy demands. These simplifications introduce potential discrepancies that could influence the overall results. Therefore, the findings should be considered as indicative, and further validation through primary data collection for precursor material synthesis or through sensitivity analysis is recommended to refine the accuracy of the assessment.

## 3 Conclusion

In summary, we successfully synthesized **P14AQ_cat_** in a Ni‐catalyzed polymerization avoiding the use of air‐ and light‐sensitive as well as expensive Ni(COD)_2_. The new synthetic route uses only catalytic amounts of NiBr_2_(PPh_3_)_2_. This route – in addition to being more cost‐efficient and less toxic – results in higher yields and polymer molecular weights, even at a scale of 2.1 mmol. A life‐cycle assessment (LCA) comparing the new and conventional polymerization methods showed that in the areas of climate change, human toxicity, and cumulative energy demand, the new method brings significant improvement.

## Conflict of Interest

The authors declare no conflict of interest.

## Supporting information



Supporting Information

## Data Availability

The data that support the findings of this study are openly available in [Zenodo] at [https://doi.org/10.5281/zenodo.15131191], reference number [15131191].
