# Supplementary material for: Synthesis of Poly(1,4‐anthraquinone) Using Catalytic Amounts of Nickel
Source: Adv Sci (Weinh). 2025 Jul 21;12(36):e06251. doi: 10.1002/advs.202506251 (PMC12463014; doi:10.1002/advs.202506251)
Supplement: Supplementary file 1 — Supporting Information [file ADVS-12-e06251-s001.pdf]

ADVANCED SCIENCE
Open Access

Supporting Information

for *Adv. Sci.*, DOI 10.1002/advs.202506251

Synthesis of Poly(1,4-anthraquinone) Using Catalytic Amounts of Nickel

*Florin Adler, Sebastián Pinto-Bautista, Christoph Lorenz, Lars Hinrichs, Marcel Weil and Birgit Esser**

Supporting Information

Synthesis of Poly(1,4-anthraquinone) Using Catalytic Amounts of Nickel

Florin Adler, Sebastián Pinto-Bautista, Christoph Lorenz, Lars Hinrichs, Marcel Weil, Birgit Esser*

Table of Contents

1. **Materials and Methods (Synthesis)** S2
2. **Materials and Methods (LCA)** S3
3. **Mass Spectrometry Data** S3
4. **Experimental Section:** S5
5. **NMR-Spectra** S7
6. **MALDI-Spectra** S9
7. **Mechanism of the $Ni(COD)_2$- and $NiBr_2(PPh_3)_2$-promoted Formation of Biaryls** ... S11
8. **Results of the LCA with the ILCD Midpoint 2011 + method** S12
9. **References** S19

1. Materials and Methods (Synthesis)

Chemicals

Chemicals were purchased from ABCR, ACROS-ORGANICS, ALFA-AESAR, BLDPHARM, JANSSEN, SIGMA-ALDRICH / MERCK or TCI and used directly without further purification unless otherwise noted.

Nickel catalysts, triphenylphosphine, and potassium iodide were purchased from BLDPHARM and SIGMA-ALDRICH/ MERCK and were dried at high vacuum (10^{-3} mbar) at 100 °C for 10 h.

Inert Working Procedures

Moisture- or oxygen-sensitive reactions were carried out in glassware that was previously dried by heating under vacuum (< 10^{-2} mbar), and standard SCHLENK techniques were applied using dry argon (Argon 5.0 by MTI INDUSTRIEGASE AG). Anhydrous DMF was obtained from an M. BRAUN solvent purification system (MB-SPS-800) and stored over activated molecular sieves (3 Å), while anhydrous 1,1,2,2-tetrachloroethane (TCE) was purchased from SIGMA-ALDRICH / MERCK (ReagentPlus®, 99%). Other solvents were used as purchased in technical or HPLC grade and used without further purification. Solvents denoted as ‘degassed’ were degassed by using three cycles of freeze-pump-thaw procedure. All polymerization reactions were set up inside a glovebox, tightly sealed using “22 mL Clear Vials” purchased from SUPELCO (27172-U) or “40 mL Clear Vials” purchased from MACHEREY-NAGEL (REF 702023).

Nuclear Magnetic Resonance Spectroscopy

NMR spectra were recorded at 300 K on a BRUKER Avance II 400 [400.1 MHz (1H), 100.6 MHz (13C)] spectrometer. Chemical shifts are reported in parts per million (ppm, δ scale). ^{1}H NMR spectra are referenced residual solvent signal of the respective solvent: $CDCl_3$: δ = 7.26 ppm. NMR spectra are referenced to the following signals: $CDCl_3$: δ = 77.16 ppm. Analysis followed first order, and the following abbreviations for multiplets are used: broad (br), singlet (s) and multiplet (m)

Mass Spectrometry

Polymer masses were acquired in positive linear mode using an Ultraflex MALDI-TOF/TOF mass spectrometer (BRUKER Daltonik, Bremen, Germany). Ionisation was achieved using a smartbeam-II laser at 1-2000 Hz repetition rates at a laser power of 50%. As internal standard a mixture of PEG 2 kDa and PEG 8 kDa was used.

MALDI-ToF sample preparation: For all MALDI measurements a DCM solution with 0.1 % TFA (stock solution) was used. For each measurement session the matrix solution was freshly prepared. For 25 samples a matrix solution consisting of DCTB (100 mg) dissolved in the stock solution (1.2 mL) was used. The polymer samples were dissolved in the stock solution (100 µL). The matrix and the sample solution were mixed in a ratio of 1:1 (30 µL each) and then pipetted onto the MALDI target (1.0 µL). For each different screening, an additional reaction with the previously improved conditions was carried out to ensure comparability within each screening. Therefore, the masses for the same reaction conditions might slightly differ.

2. Materials and Methods (LCA)

Goal and scope definition

The goal of this study, as previously mentioned, is to quantify the potential environmental advantages and disadvantages of the newly developed Ni-catalyzed **P14AQ** synthesis route compared to the conventional YAMAMOTO method. The system boundaries are defined as cradle-to-gate, meaning that the impacts associated with resource extraction, pre-chain processes, and **P14AQ** production are considered. The functional unit, which serves as the reference for quantifying environmental impacts, is defined as 1 g of **P14AQ**.

Life cycle inventory analysis

The inventory analysis, which involves creating datasets that describe the inputs and outputs of the system, was conducted by analysing the chemical reactions described in the literature for the reference process.[11] For the synthesis route developed in this study, primary data on material and energy demand was collected directly from laboratory experiments. Data for precursors and other compounds was sourced, where available, from the commercial database Ecoinvent 3.10 (ecoinvent.org). Materials not found in this database were modelled based on literature sources, stoichiometric calculations and approximations to reference materials. It is important to note that lab-scale processes are typically characterized by small production rates, low equipment utilization, and limited attention to resource efficiency, often leading to significant overestimations of material and energy demand. In particular, overestimations of energy use could overshadow the contributions of reactants and other material flows, complicating the interpretation of results. To mitigate this issue, energy flows such as heat and electricity were estimated theoretically, assuming larger production scales for the stirring and drying steps, as described by SOM and coworkers.[34] The energy matrix used in the model corresponds to the average European mix for both heat and electricity.

Life cycle impact assessment

The selected impact-assessment method is the International Reference Life Cycle Data System (ILCD), specifically ILCD Midpoint 2011 +, as it aligns with EU sustainability initiatives, such as the Product Environmental Footprint (PEF) from the European Commission.[35] This method also provides a harmonized approach for characterizing environmental impacts at the midpoint level, including a set of 16 midpoint level indicators relating to different environmental impact categories of concern.[36]

3. Mass Spectrometry Data

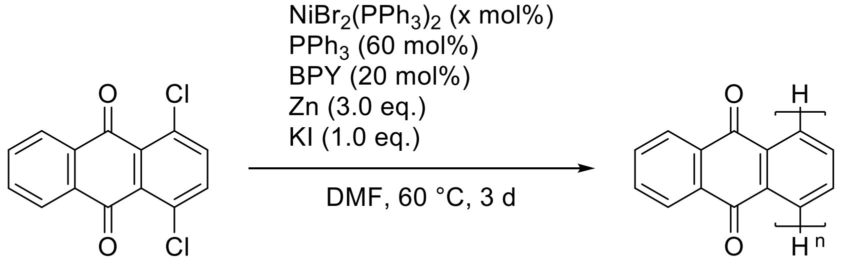

DC14AQ **P14AQ**

***Scheme S1**: Reaction conditions for the catalyst amount screening with $NiBr_2(PPh_3)_2$ (Table S1).*

***Table S1**: Overview of the results of the Ni-catalyzed polymerization of **14DCAQ** to **P14AQ$_{cat}$** by increasing the catalyst amount ($NiBr_2(/PPh_3)_2$) (Scheme 1).*

Entry	Amount of catalyst [mol%]	$\bar{M}_n$ [g mol^{-1}]	$\bar{M}_w$[g mol^{-1}]	Đ	DP
1	10	3,460	4,400	1.27	17
2	20	6,310	6,760	1.07	31
3	25	5,510	6,460	1.17	27
4	30	5,980	6,370	1.09	29
5	35	5,510	6,340	1.15	27
6	40	5,270	5,610	1.06	26
7	60	4,540	5,030	1.11	22
8	80	5,230	5,370	1.03	26
9	100	4,920	5,310	1.08	24

The number average molar mass ($\bar{M}_n$), mass average molar mass ($\bar{M}_w$), dispersity (Đ) and the degree of polymerization (DP) were calculated with BRUKER MS software POLYTOOL.

***Table S2**: Overview of the results variating the phosphine amount.*

Entry	Amount of PPh_3 [mol%]	$\bar{M}_n$ [g mol^{-1}]	$\bar{M}_w$ [g mol^{-1}]	Đ	DP
1	0	5,970	6,720	1.12	29
2	10	5,650	6,420	1.14	28
3	20	5,620	6,470	1.15	27
4	30	5,260	6,410	1.22	26
5	40	5,450	6,200	1.14	27
6	50	5,910	6,610	1.11	29
7	60	5,500	6,360	1.15	27

The number average molar mass ($\bar{M}_n$), mass average molar mass ($\bar{M}_w$), dispersity (Đ) and the degree of polymerization (DP) were calculated with BRUKER MS software POLYTOOL.

***Table S3**: Overview of the results of variating the ligand with 20 mol% each.*

Entry	Ligand	$\bar{M}_n$ [g mol^{-1}]	$\bar{M}_w$[g mol^{-1}]	Đ	DP
1	2,2‘-bipyridine	5,980	6,250	1.04	29
2	4,4‘-dimethyl-2,2‘-bipyridine	4,780	5,010	1.05	24
3	phenanthroline	2,780	3,150	1,14	14
4	4,7-diphenyl-1,10-phenanthroline	-	-	-	-

The number average molar mass ($\bar{M}_n$), mass average molar mass ($\bar{M}_w$), dispersity (Đ) and the degree of polymerization (DP) were calculated with BRUKER MS software POLYTOOL.

4. Experimental Section:

1,4-Dichloroanthraquinone (14DCAQ)

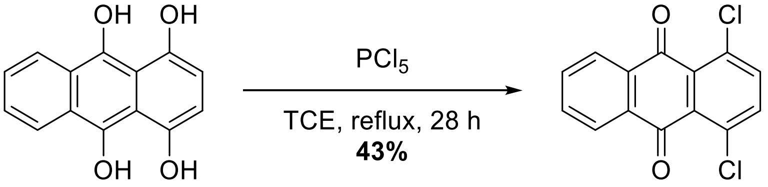

The synthesis of **14DCAQ** was performed following a modified procedure by MEADOR and coworkers.[37]

PCl_5 (50.0 g, 240 mmol, 6.0 eq.) was dissolved in anhydrous 1,1,2,2-tetrachloroethane (TCE, 30 mL). Leucoquarizarine (9.69 g, 40.0 mmol) was slowly added, and the suspension was refluxed for 28 h. After cooling to room temperature, the mixture was slowly quenched with methanol (30 mL). The precipitate was filtered and washed with methanol. The crude product was dissolved in 1-pentanol (40 mL) over 3 h at 160 °C. The solution was cooled to room temperature and stored at −20 °C over night. The resulting precipitate was filtered, washed with methanol, treated with a mortar and pestle and dried at 100 °C at high vacuum to afford **14DCAQ** (4.80 g, 17.3 mmol, 43%) as golden crystalline solid.

^{1}H NMR (400 MHz, $CDCl_3$): δ 8.23–8.17 (m, 2 H), 7.82–7.76 (m, 2 H), 7.68 (s, 2 H); ^{13}C NMR: (101 MHz, $CDCl_3$): δ 181.9, 137.3, 134.4, 134.2, 133.8, 132.3, 127.1.

State-of-the-art synthesis of Poly(1,4-anthraquinone) (P14AQ$_{YAM}$) using stoichiometric amounts of Ni(0)

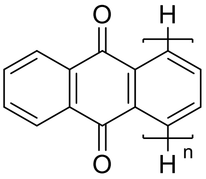

The synthesis of **P14AQ$_{YAM}$** was performed following a modified procedure by CHEN and coworkers.[17]

14DCAQ (582 mg, 2.1 mmol), $Ni(COD)_2$ (751 mg, 2.7 mmol, 1.3 eq.), 2,2'-bipyridine (426 mg, 2.7 mmol, 1.3 eq.) and degassed cyclooctadiene (0.26 mL, 229 mg, 2.1 mmol, 1.0 eq.) were suspended in anhydrous degassed DMF (5.0 mL), and the resulting suspension was stirred at 60 °C for 69 h. The mixture was poured into an aq. solution of HCl (1 M, 50 mL) and the resulting precipitate was filtered, washed with an aq. solution of HCl (1 M), DMF, deionized water, methanol and cyclohexane and dried at 80 °C at high vacuum for 10 h to afford **P14AQ$_{YAM}$** (380 mg, 1.83 mmol, 87%) as a yellow solid.

^{1}H NMR (400 MHz, $CDCl_3$): δ 8.30 (br., 1 H), 8.10 (br. 1 H), 7.89 (br. 1 H), 7.72 (br. 3 H); MALDI-ToF: $\bar{M}_n$ = 4445, $\bar{M}_w$ = 4894, $Đ$ = 1.10, DP = 22%.

Optimized procedure for the Ni-catalyzed synthesis of Poly(1,4-anthraquinone) ($P14AQ_{cat}$)

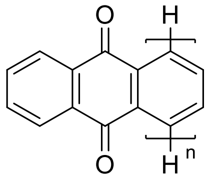

Small-scale reaction (scale used for optimization):

14DCAQ (83 mg, 0.30 mmol), $NiBr_2(PPh_3)_2$ (45 mg, 0.06 mmol, 20 mol%), 2,2'-bipyridine (9 mg, 0.06 mmol, 20 mol%), KI (50 mg, 0.30 mmol, 1.0 eq.) and Zn powder (58 mg, 0.90 mmol, 3.0 eq.) were suspended in anhydrous degassed DMF (5.0 mL), and the resulting suspension was stirred at 60 °C for 69 h. The mixture was poured into an aq. solution of HCl (1 M, 50 mL) and stirred overnight to quench the excess of Zn powder. The resulting precipitate was separated from the solvent by centrifugation (5 min, 6000 rpm) and decanting the solvent. The solid was resuspended in an aq. solution of HCl (1 M, 2 × 30 mL) and separated by centrifugation (5 min, 6000 rpm) and decanting the solvent. This step was repeated once more with an aq. solution of HCl (1 M, 30 mL), DMF, (2 × 30 mL) deionized water (2 × 30 mL), methanol (3 × 30 mL) and cyclohexane (30 mL) and dried at 80 °C at high vacuum for 10 h to afford **$P14AQ_{cat}$** (61.2 mg, 0.29 mmol, 98%) as a yellow solid.

^{1}H NMR (400 MHz, $CDCl_3$): δ 8.30 (br., 1 H), 8.10 (br. 1 H), 7.89 (br. 1 H), 7.72 (br. 3 H); MALDI-ToF: $\bar{M}_n$ = 6340 g mol^{-1}, $\bar{M}_w$= 6791 g mol^{-1}, $Đ$ = 1.07, DP = 31%.

Scaled up reaction:

14DCAQ (582 mg, 2.10 mmol), $NiBr_2(PPh_3)_2$ (312 mg, 0.42 mmol, 20 mol%), 2,2'-bipyridine (66 mg, 0.42 mmol, 20 mol%), KI (350 mg, 2.10 mmol, 1.0 eq.) and Zn powder (412 mg, 6.30 mmol, 3.0 eq.) were suspended in anhydrous degassed DMF (35.0 mL), and the resulting suspension was stirred at 60 °C for 69 h. The mixture was slowly poured into an aq. solution of HCl (1 M, 350 mL) and stirred overnight to quench the excess of Zn powder. The resulting precipitate was filtered, washed with an aq. solution of HCl (1 M), DMF, deionized water, methanol and cyclohexane and dried at 80 °C at high vacuum for 10 h to afford **$P14AQ_{cat}$** (405 mg, 1.96 mmol, 93%) as a yellow solid.

^{1}H NMR (400 MHz, $CDCl_3$): δ 8.30 (br., 1 H), 8.10 (br. 1 H), 7.89 (br. 1 H), 7.72 (br. 3 H); MALDI-ToF: $\bar{M}_n$ = 5792, $\bar{M}_w$ = 6326, $Đ$ = 1.09, DP = 28%.

5. NMR-Spectra

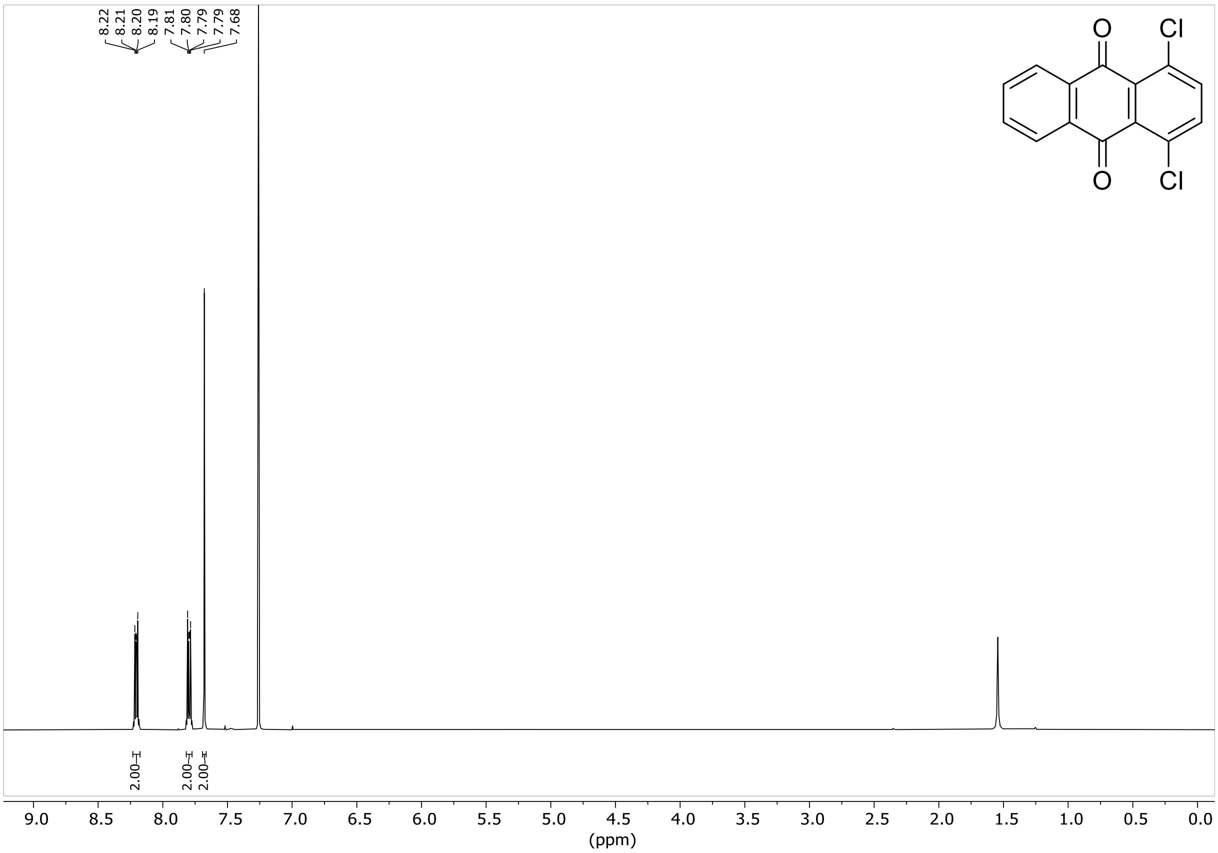

***Figure S1**: 400 MHz ^{1}H NMR spectrum of 1,4-dichloroanthracene-9,10-dione (**14DCAQ**) in $CDCl_3$*

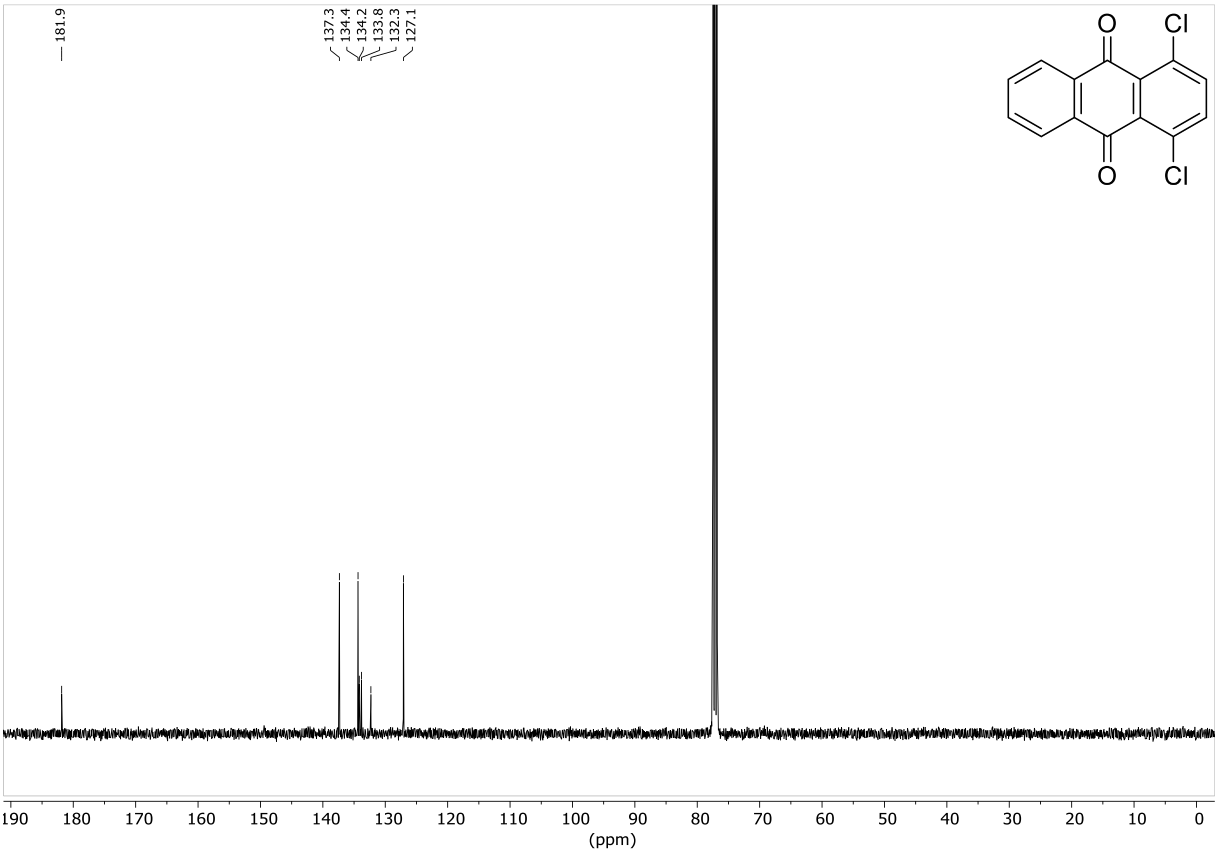

***Figure S2**: 101 MHz ^{13}C NMR spectrum of 1,4-dichloroanthracene-9,10-dione (**14DCAQ**) in $CDCl_3$.*

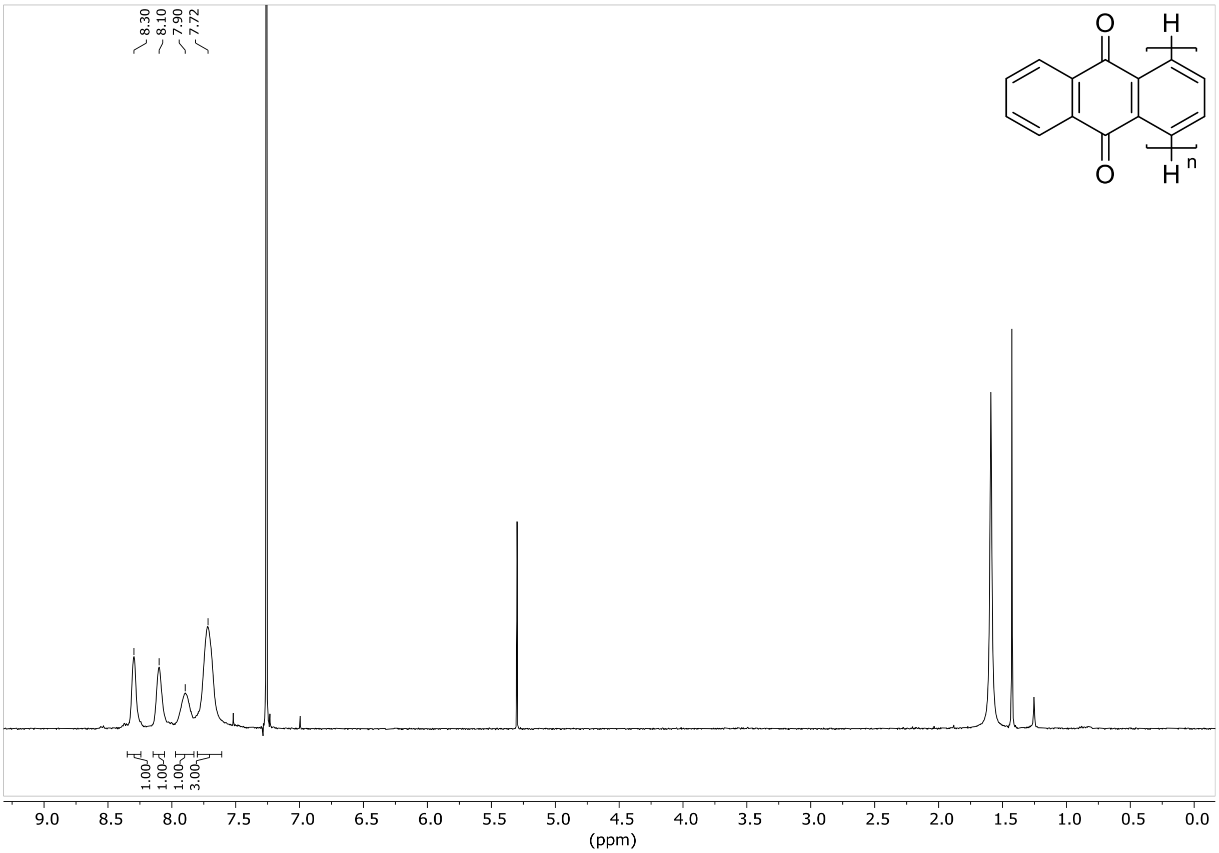

***Figure S3**: 400 MHz ^{1}H NMR spectrum of* Poly(1,4-anthraquinone) *(**$P14AQ_{YAM}$**) in $CDCl_3$.*

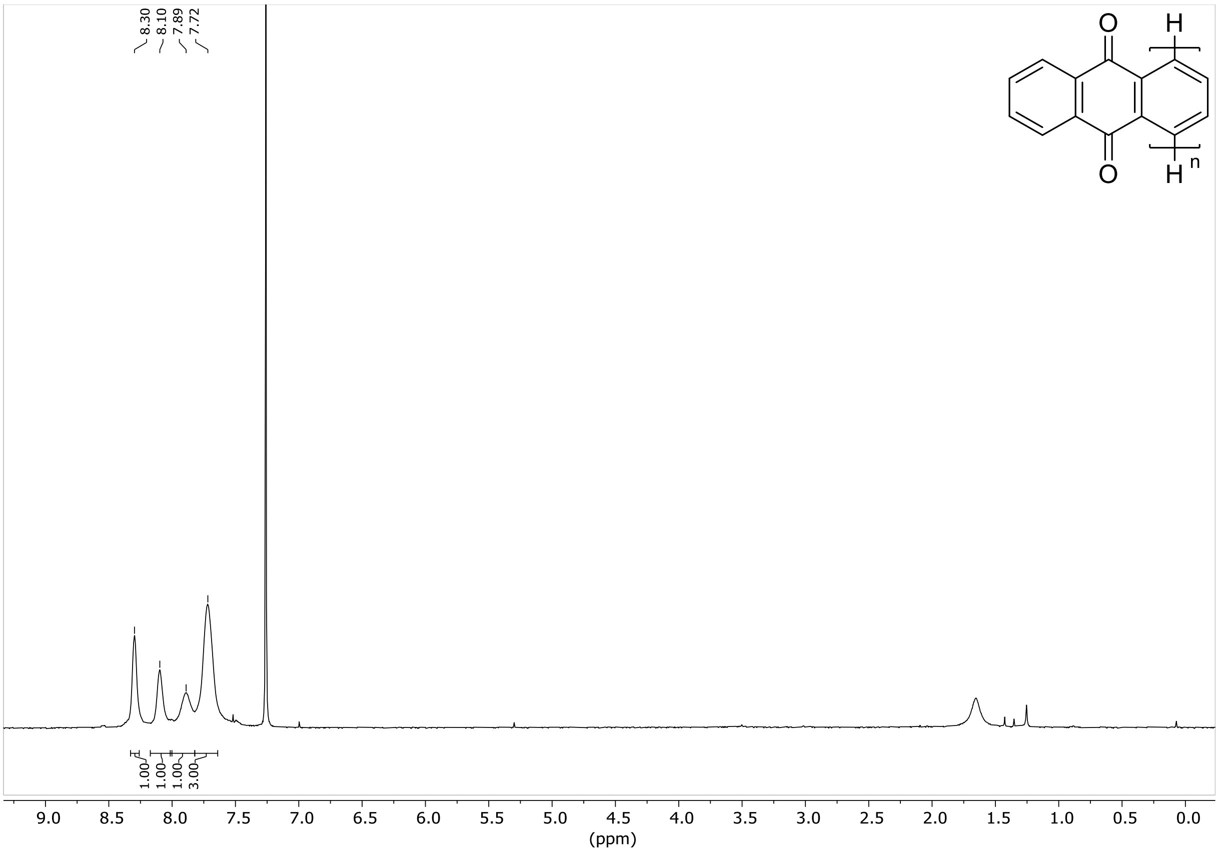

***Figure S4**: 400 MHz ^{1}H NMR spectrum of* Poly(1,4-anthraquinone) *(**$P14AQ_{cat}$**) in $CDCl_3$ (scale up).*

6. MALDI-Spectra

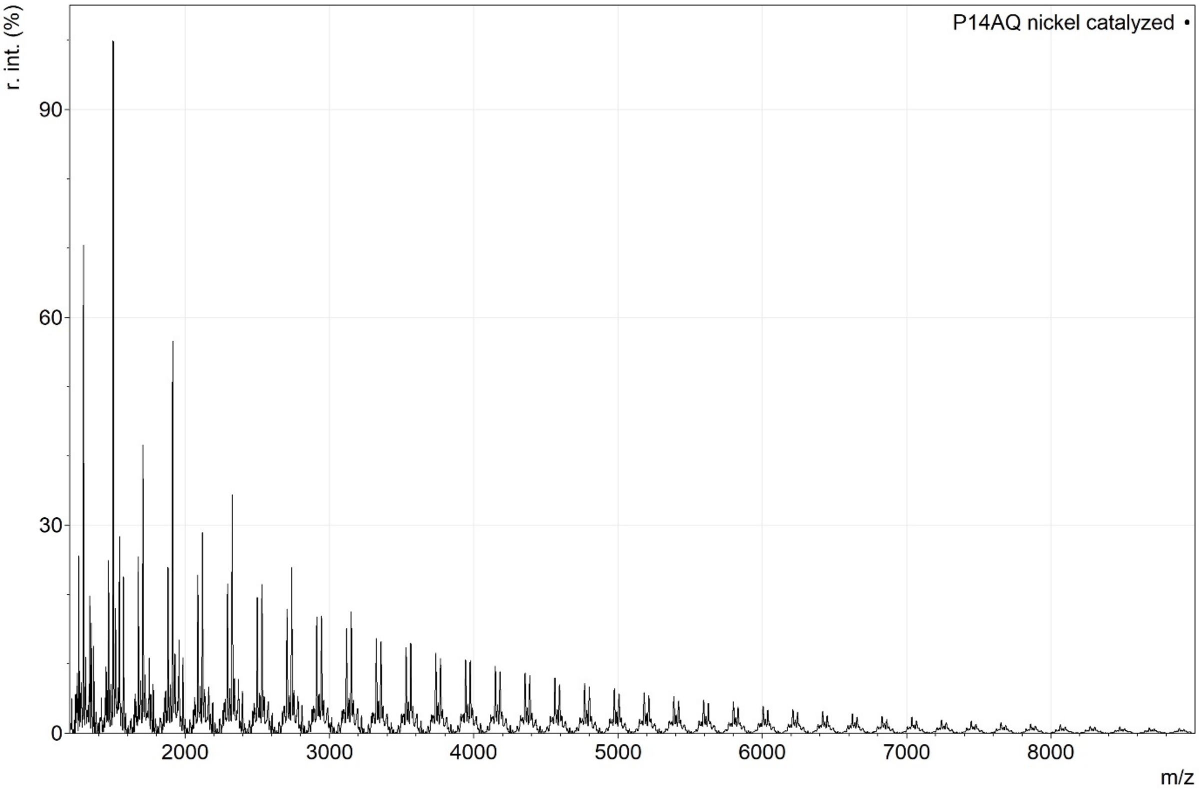

Figure S5*: MALDI-ToF spectra of Poly(1,4-anthraquinone) (**P14AQ**) with $NiCl_2$ (10 mol%) as catalyst.*

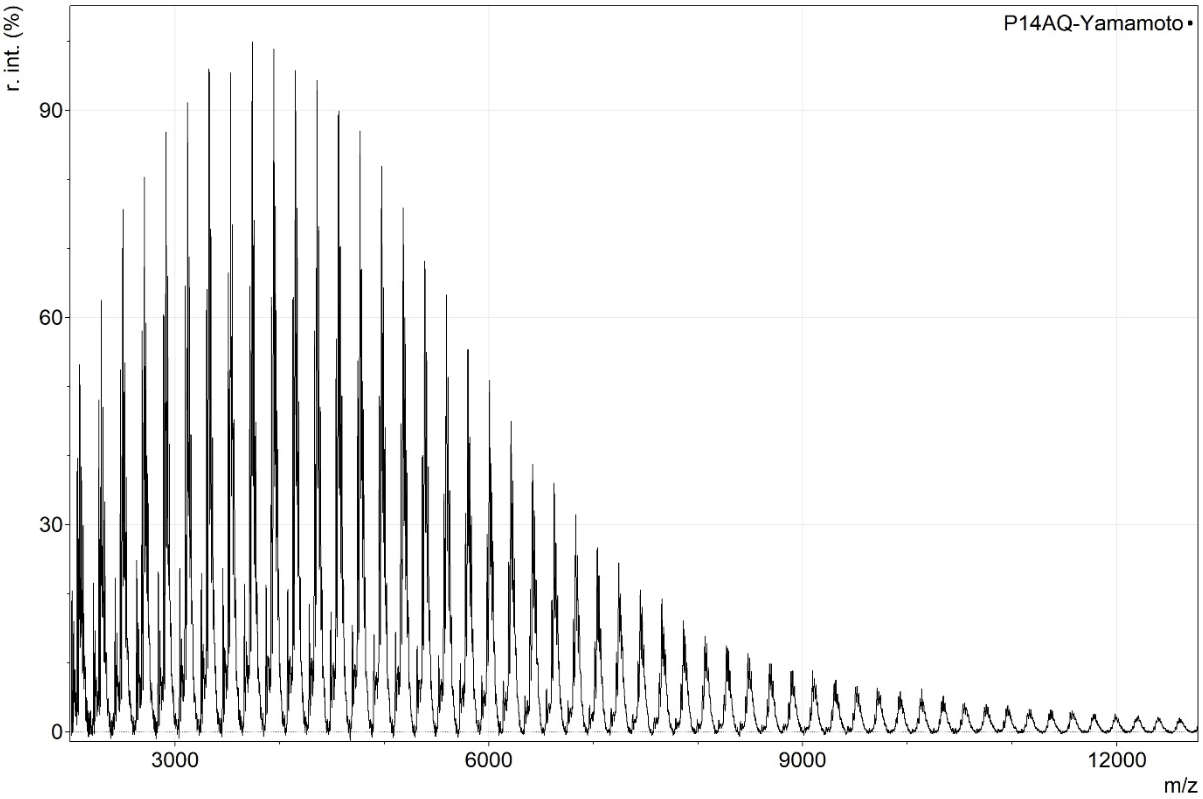

Figure S6*: MALDI-ToF spectra of Poly(1,4-anthraquinone) (**$P14AQ_{YAM}$**).*

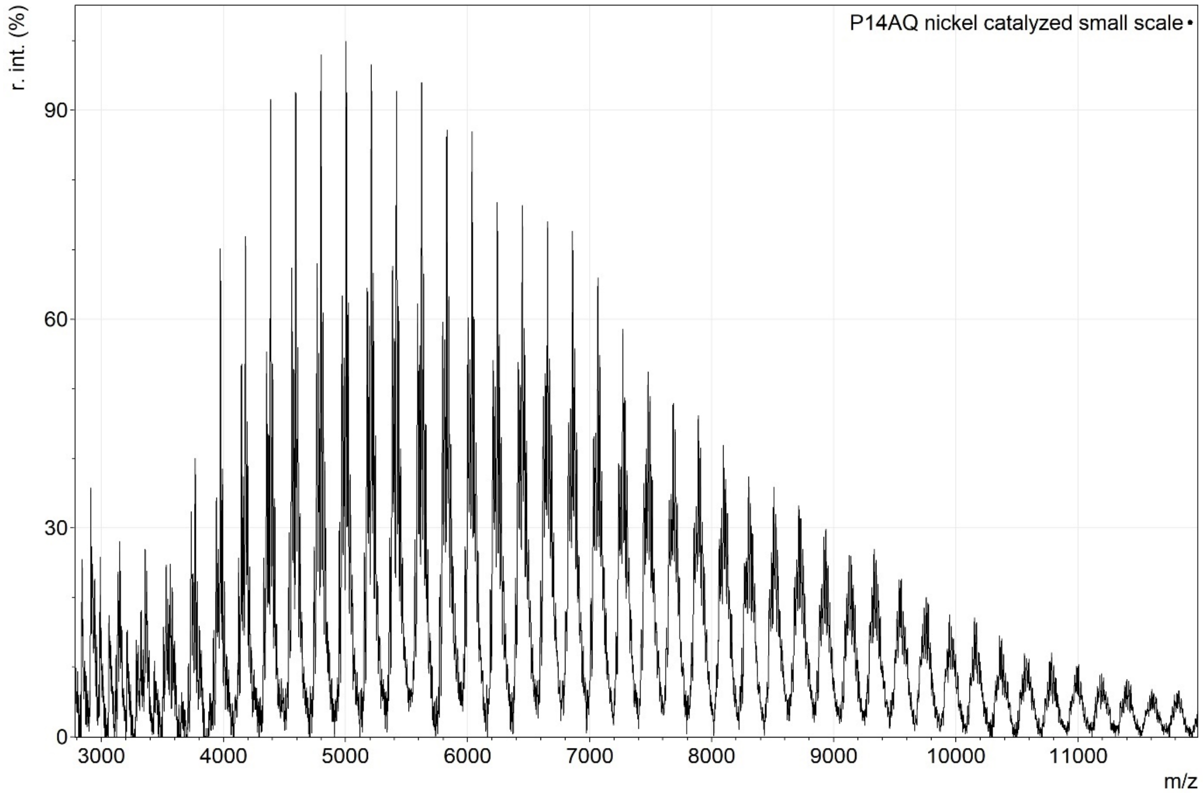

Figure S7: *MALDI-ToF spectra of Poly(1,4-anthraquinone) (**P14AQ$_{cat}$**) (small scale).*

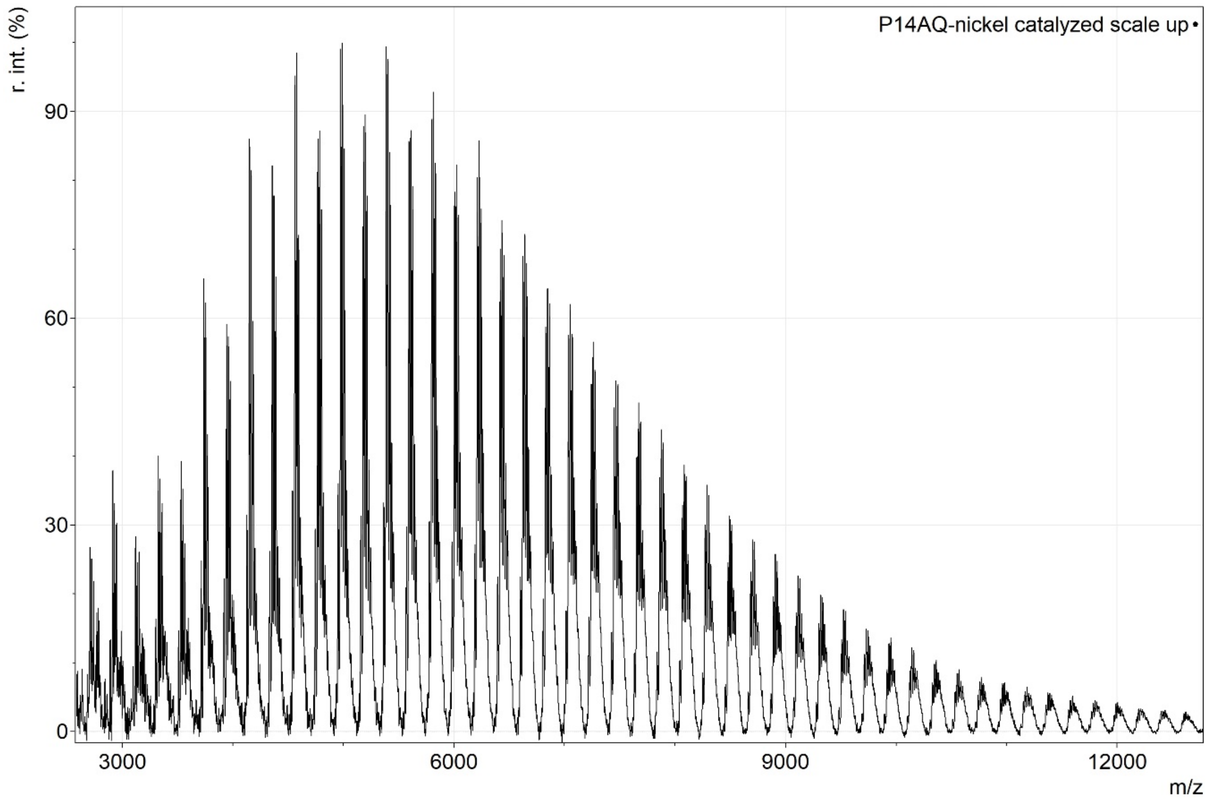

Figure S8: *MALDI-ToF spectra of Poly(1,4-anthraquinone) (**P14AQ$_{cat}$**) (scale up).*

7. Mechanism of the $Ni(COD)_2$- and $NiBr_2(PPh_3)_2$-promoted Formation of Biaryls

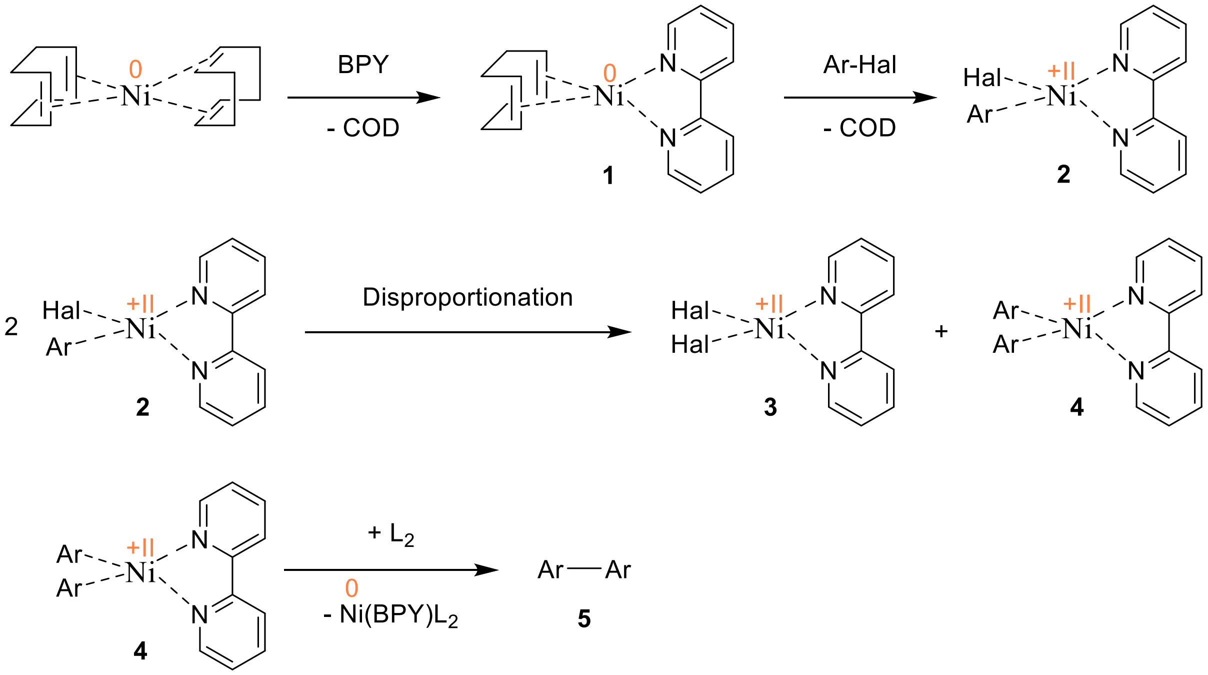

***Scheme S2**: Proposed mechanism of the $Ni(COD)_2$-promoted formation of biaryls with L as an available ligand.*[38]

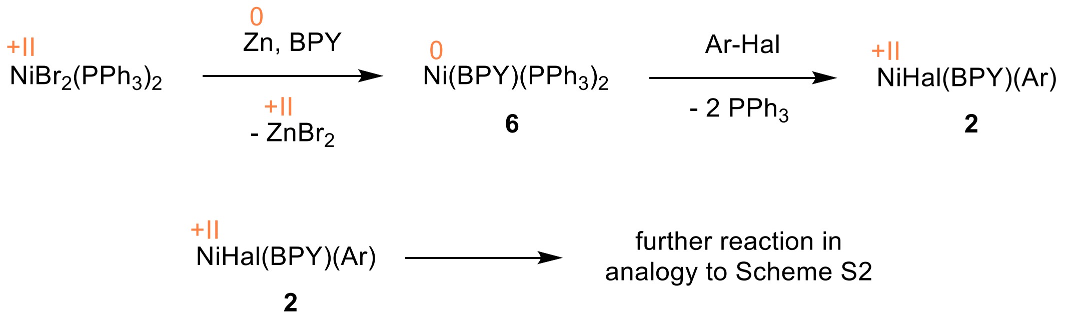

***Scheme S3**: Adjusted mechanism of the $NiBr_2(PPh_3)_2$-catalyzed formation of biaryls.*

8. Results of the LCA with the ILCD Midpoint 2011 + method

Table S4: *Full list of results in all impact categories of the ILCD method.*

Entry	Impact category	YAMAMOTO	Ni-catalyzed	Unit
1	Acidification	1.69×10^{-3}	1.70×10^{-3}	molc H^+ eq
2	Climate change	3.71×10^{-1}	3.38×10^{-1}	kg CO_2 eq
3	Freshwater ecotoxicity	5.89	5.86	CTUe
4	Freshwater eutrophication	9.09×10^{-5}	9.97×10^{-5}	kg P eq
5	Human toxicity, cancer effects	9.30×10^{-8}	7.71×10^{-8}	CTUh
6	Human toxicity, non-cancer effects	8.02×10^{-8}	8.97×10^{-8}	CTUh
7	Ionizing radiation E (interim)	1.42×10^{-7}	1.20×10^{-7}	CTUe
8	Ionizing radiation HH	4.91×10^{-2}	4.58×10^{-2}	kBq U^{235} eq
9	Land use	2.84×10^{-1}	2.63×10^{-1}	kg C deficit
10	Marine eutrophication	3.08×10^{-4}	2.29×10^{-4}	kg N eq
11	Mineral, fossil & ren resource depletion	1.58×10^{-5}	2.34×10^{-5}	kg Sb eq
12	Ozone depletion	1.37×10^{-8}	1.49×10^{-8}	kg CFC-11 eq
13	Particulate matter	1.18×10^{-4}	1.16×10^{-4}	kg PM2.5 eq
14	Photochemical ozone formation	1.29×10^{-3}	1.14×10^{-3}	kg NMVOC eq
15	Terrestrial eutrophication	4.36×10^{-3}	4.10×10^{-3}	molc N eq
16	Water resource depletion	2.28×10^{-1}	2.05×10^{-1}	m^3 water eq

Life cycle inventories

- Flows and providers are indicated as presented in the Ecoinvent 3.10 database
- Values of mass and energy flows based on stoichiometry are calculated considering 80% reaction efficiencies.
- Production processes are assumed to take place in Europe.

Table S5: *LCI of Nickel hydroxide. Adapted for nickel cobalt manganese hydroxide.* [39]

Flow	Amount	Unit	Provider
Inputs			
chemical factory, organics	4.00×10^{-10}	Item(s)	chemical factory construction, organics | chemical factory, organics | Cutoff, U - RER
nickel sulfate	1.68	kg	market for nickel sulfate | nickel sulfate | Cutoff, U - GLO
sodium hydroxide, without water, in 50% solution state	0.88	kg	market for sodium hydroxide, without water, in 50% solution state | sodium hydroxide, without water, in 50% solution state | Cutoff, U - RER
Outputs			
Nickel hydroxide	1.00	kg	-

Table S6: *LCI of Nickel oxide.*[40]

Flow	Amount	Unit	Provider
Inputs			
chemical factory, organics	4.00×10^{-10}	Item(s)	chemical factory construction, organics | chemical factory, organics | Cutoff, U - RER

heat, district or industrial, other than natural gas	2.70	MJ	market for heat, district or industrial, other than natural gas | heat, district or industrial, other than natural gas | Cutoff, U - Europe without Switzerland
Nickel hydroxide	1.25	kg	Table S5
		Outputs	
Nickel oxide	1.00	kg	-
Water	0.25	kg	-

***Table S7**: LCI of Nickel nitrate hexahydrate.*[41]

Flow	Amount	Unit	Provider
		Inputs	
chemical factory, organics	4.00×10^{-10}	Item(s)	chemical factory construction, organics | chemical factory, organics | Cutoff, U - RER
heat, district or industrial, other than natural gas	0.43	MJ	market group for heat, district or industrial, other than natural gas | heat, district or industrial, other than natural gas | Cutoff, U - RER
Nickel oxide	321	g	Table S6
nitric acid, without water, in 50% solution state	543	g	market for nitric acid, without water, in 50% solution state | nitric acid, without water, in 50% solution state | Cutoff, U - CN
tap water	388	g	market for tap water | tap water | Cutoff, U - Europe without Switzerland
		Outputs	
Nickel nitrate hexahydrate	1.00	kg	-

***Table S8**: LCI of* Acetylacetone. [42-43]

Flow	Amount	Unit	Provider
		Inputs	
acetic acid, without water, in 98% solution state (substituting Ketene)	525	g	market for acetic acid, without water, in 98% solution state | acetic acid, without water, in 98% solution state | Cutoff, U - GLO
acetone, liquid	725	g	market for acetone, liquid | acetone, liquid | Cutoff, U - RER
chemical factory, organics	4.00×10^{-10}	Item(s)	chemical factory construction, organics | chemical factory, organics | Cutoff, U - RER
heat, district or industrial, other than natural gas	1.00	MJ	market for heat, district or industrial, other than natural gas | heat, district or industrial, other than natural gas | Cutoff, U - Europe without Switzerland
		Outputs	
Acetylacetone	1.00	kg	-

***Table S9**: LCI of Nickel(II) bis(acetylacetonate).*[44]

Flow	Amount	Unit	Provider
		Inputs	
Acetylacetone	866	g	Table S8

chemical factory, organics	4.00 × 10^{-10}	Item(s)	chemical factory construction, organics | chemical factory, organics | Cutoff, U - RER
heat, district or industrial, other than natural gas	1133	KJ	market for heat, district or industrial, other than natural gas | heat, district or industrial, other than natural gas | Cutoff, U - Europe without Switzerland
Nickel nitrate hexahydrate	1404	g	Table S7
sodium hydroxide, without water, in 50% solution state	395	g	market for sodium hydroxide, without water, in 50% solution state | sodium hydroxide, without water, in 50% solution state | Cutoff, U - RER
tap water	178	g	market for tap water | tap water | Cutoff, U - Europe without Switzerland
		Outputs	
Nickel(II) bis(acetylacetonate)	1	Kg	-
sodium nitrate	669	g	-

Table S10*: LCI of Triethylaluminium. Extracted from.* [45]

Flow	Amount	Unit	Provider
		Inputs	
aluminium, primary, ingot	237	g	market for aluminium, primary, ingot | aluminium, primary, ingot | Cutoff, U - IAI Area, EU27 & EFTA
chemical factory, organics	4.00 × 10^{-10}	Item(s)	chemical factory construction, organics | chemical factory, organics | Cutoff, U - RER
electricity, medium voltage	382	KJ	market group for electricity, medium voltage | electricity, medium voltage | Cutoff, U - ENTSO-E
ethylene	778	g	market for ethylene | ethylene | Cutoff, U - RER
heat, district or industrial, other than natural gas	2.29	MJ	market for heat, district or industrial, other than natural gas | heat, district or industrial, other than natural gas | Cutoff, U - Europe without Switzerland
hydrogen, gaseous, low pressure	27.8	g	market for hydrogen, gaseous, low pressure | hydrogen, gaseous, low pressure | Cutoff, U - RER
		Outputs	
Triethylaluminium	1.00	kg	-
ethylene	40.7	g	-
Hydrogen	1.45	g	-

Table S11*: LCI of COD (cyclooctadiene).*[46]

Flow	Amount	Unit	Provider
		Inputs	
butadiene	1.25	kg	market for butadiene | butadiene | Cutoff, U - RER
chemical factory, organics	4.00 × 10^{-10}	Item(s)	chemical factory construction, organics | chemical factory, organics | Cutoff, U - RER
heat, district or industrial, natural gas	0.75	MJ	market for heat, district or industrial, natural gas | heat, district or industrial, natural gas | Cutoff, U - Europe without Switzerland

Outputs			
COD (cyclooctadiene)	1.00	kg	-
butadiene	0.25	kg	market for butadiene | butadiene | Cutoff, U - RER

***Table S12**: LCI of Ni(COD)$_2$ (Nickel bis-cyclooctadiene).*[47]

Flow	Amount	Unit	Provider
Inputs			
chemical factory, organics	4.00×10^{-10}	Item(s)	chemical factory construction, organics | chemical factory, organics | Cutoff, U - RER
COD (cyclooctadiene)	983	g	Table S11
heat, district or industrial, other than natural gas	1.00	MJ	market group for heat, district or industrial, other than natural gas | heat, district or industrial, other than natural gas | Cutoff, U - RER
Nickel(II) bis(acetylacetonate)	1.17	kg	Table S9
Triethylaluminium	1.04	kg	Table S10
Outputs			
Nickel bis-cyclooctadiene (Ni(COD)$_2$)	1.00	kg	-
ethane	109	g	-
ethylene	101	g	-
hazardous waste, for incineration	1.34	kg	market for hazardous waste, for incineration | hazardous waste, for incineration | Cutoff, U - Europe without Switzerland

***Table S13**: LCI of NiBr$_2$ (Nickel bromide). Based on the stoichiometric reaction of Nickel metal with Bromine.* [48]

Flow	Amount	Unit	Provider
Inputs			
bromine	733	g	market for bromine | bromine | Cutoff, U - GLO
chemical factory, organics	4.00×10^{-10}	Item(s)	chemical factory construction, organics | chemical factory, organics | Cutoff, U - RER
nickel, class 1	216	g	market for nickel, class 1 | nickel, class 1 | Cutoff, U - GLO
tap water	248	g	market for tap water | tap water | Cutoff, U - Europe without Switzerland
Outputs			
Nickel bromide (NiBr$_2$)	1.00	kg	-

***Table S14**: LCI of PPh$_3$ (Triphenylphosphine).* [49]

Flow	Amount	Unit	Provider
Inputs			
chemical factory, organics	4.00×10^{-10}	Item(s)	chemical factory construction, organics | chemical factory, organics | Cutoff, U - RER
heat, district or industrial, natural gas	4.90	MJ	market for heat, district or industrial, natural gas | heat, district or industrial, natural gas | Cutoff, U - Europe without Switzerland

monochlorobenzene	1.62	kg	market for monochlorobenzene | monochlorobenzene | Cutoff, U - RER
phosphorus trichloride	1.00	kg	market for phosphorus trichloride | phosphorus trichloride | Cutoff, U - GLO
sodium	0.66	kg	market for sodium | sodium | Cutoff, U - GLO
		Outputs	
Triphenylphosphine (PPh_3)	1.00	kg	-

***Table S15**: LCI of $NiBr_2(PPh_3)_2$ (Nickel(II) bromide bis(triphenylphosphine)).*[50]

Flow	Amount	Unit	Provider
		Inputs	
chemical factory, organics	4.00×10^{-10}	Item(s)	chemical factory construction, organics | chemical factory, organics | Cutoff, U - RER
heat, district or industrial, other than natural gas	0.45	MJ	market for heat, district or industrial, other than natural gas | heat, district or industrial, other than natural gas | Cutoff, U - Europe without Switzerland
Nickel bromide ($NiBr_2$)	0.59	kg	Table S13
Triphenylphosphine (PPh_3)	0.99	kg	Table S14
		Outputs	
Nickel(II) bromide bis(triphenylphosphine) ($NiBr_2(PPh_3)_2$)	1.00	kg	-
Water	81.5	g	-

***Table S16**: LCI of **14DCAQ** (1,4-dicholoanthraquinone).*[51]

Flow	Amount	Unit	Provider
		Inputs	
chemical factory, organics	4.00×10^{-10}	Item(s)	chemical factory construction, organics | chemical factory, organics | Cutoff, U - RER
heat, district or industrial, natural gas	9.00	MJ	market for heat, district or industrial, natural gas | heat, district or industrial, natural gas | Cutoff, U - Europe without Switzerland
p-dichlorobenzene	663	g	market for p-dichlorobenzene | p-dichlorobenzene | Cutoff, U - RER
phthalic anhydride	668	kg	market for phthalic anhydride | phthalic anhydride | Cutoff, U - GLO
		Outputs	
**14DCAQ** (1,4-dicholoanthraquinone)	1.00	kg	-
hydrochloric acid, without water, in 30% solution state	263	g	market for hydrochloric acid, without water, in 30% solution state | hydrochloric acid, without water, in 30% solution state | Cutoff, U - RER
hazardous waste, for incineration	66.0	g	market for hazardous waste, for incineration | hazardous waste, for incineration | Cutoff, U - Europe without Switzerland

***Table S17**: LCI of BPY (2,2´-bipyridine).*[52]

Flow	Amount	Unit	Provider
		Inputs	
chemical factory, organics	4.00×10^{-10}	Item(s)	chemical factory construction, organics | chemical factory, organics | Cutoff, U - RER
heat, district or industrial, natural gas	9.60	MJ	market for heat, district or industrial, natural gas | heat, district or industrial, natural gas | Cutoff, U - Europe without Switzerland
pyridine	1.25	kg	pyridine production, Chichibabin process | pyridine | Cutoff, U - RER
		Outputs	
BPY (2,2´bipyridine)	1.00	kg	-
pyridine	0.25	kg	market for pyridine | pyridine | Cutoff, U - GLO

***Table S18**: LCI of KI (Potassium iodide).* [53]

Flow	Amount	Unit	Provider
		Inputs	
chemical factory, organics	4.00×10^{-10}	Item(s)	chemical factory construction, organics | chemical factory, organics | Cutoff, U - RER
heat, district or industrial, natural gas	1.00	MJ	market for heat, district or industrial, natural gas | heat, district or industrial, natural gas | Cutoff, U - Europe without Switzerland
iodine	1.02	kg	market for iodine | iodine | Cutoff, U - GLO
potassium hydroxide	0.45	kg	market for potassium hydroxide | potassium hydroxide | Cutoff, U - GLO
		Outputs	
Potassium iodide (KI)	1.00	kg	-
hazardous waste, for incineration	398	g	-
Water	72	g	-

***Table S19**: LCI of Poly(1,4-anthraquinone) (**P14AQ**) synthesis [YAMAMOTO].*[11]

Flow	Amount	Unit	Provider
		Inputs	
BPY (2,2´-bipyridine)	427	mg	Table S17
COD (cyclooctadiene)	227	mg	Table S11
**14DCAQ** (1,4-dicholoanthraquinone)	582	mg	Table S16
electricity, medium voltage	0.03	Wh	market group for electricity, medium voltage | electricity, medium voltage | Cutoff, U - ENTSO-E
heat, district or industrial, natural gas	1.31	Wh	market group for heat, district or industrial, natural gas | heat, district or industrial, natural gas | Cutoff, U - RER
hydrochloric acid, without water, in 30% solution state	0.64	g	market for hydrochloric acid, without water, in 30% solution state | hydrochloric acid, without water, in 30% solution state | Cutoff, U - RER

N,N-dimethylformamide	33.0	g	N,N-dimethylformamide production, direct synthesis | N,N-dimethylformamide | Cutoff, U - RER
Nickel bis-cyclooctadiene ($Ni(COD)_2$)	751	mg	Table S12
Outputs			
Poly(1,4-anthraquinone) (**P14AQ**)	372	mg	-

Table S20: *LCI of Poly(1,4-anthraquinone) (**P14AQ**) synthesis [Ni-catalyzed].*

Flow	Amount	Unit	Provider
Inputs			
BPY (2,2-bipyridine)	65.6	mg	Table S17
**14DCAQ** (1,4-dicholoanthraquinone)	582	mg	Table S16
electricity, medium voltage	0.12	Wh	market group for electricity, medium voltage | electricity, medium voltage | Cutoff, U - ENTSO-E
heat, district or industrial, other than natural gas	1.43	Wh	market for heat, district or industrial, other than natural gas | heat, district or industrial, other than natural gas | Cutoff, U - Europe without Switzerland
hydrochloric acid, without water, in 30% solution state	10.9	g	market for hydrochloric acid, without water, in 30% solution state | hydrochloric acid, without water, in 30% solution state | Cutoff, U - RER
N,N-dimethylformamide	33.0	g	N,N-dimethylformamide production, direct synthesis | N,N-dimethylformamide | Cutoff, U - RER
Nickel(II) bromide bis(triphenylphosphine) ($NiBr_2(PPh_3)_2$)	312	mg	Table S15
Potassium iodide (KI)	350	mg	Table S18
zinc	412	mg	market for zinc | zinc | Cutoff, U - GLO
Outputs			
Poly(1,4-anthraquinone) (P14AQ)	412	mg	-

9. References

[11] Z. Song, Y. Qian, M. L. Gordin, D. Tang, T. Xu, M. Otani, H. Zhan, H. Zhou, D. Wang, *Angew. Chemie* **2015**, *127*, 14153.

[17] Y. Li, Y. Lu, Y. Ni, S. Zheng, Z. Yan, K. Zhang, Q. Zhao, J. Chen, *J. Am. Chem. Soc.* **2022**, *144*, 8066.

[34] F. Piccinno, R. Hischier, S. Seeger, C. Som, *J. Clean. Prod.* **2016**, *135*, 1085.

[35] European Commission, *PEFCR - Prod. Environ. Footpr. Categ. Rules* **2021**, 1.

[36] European Commission, *ILCD Handbook: General Guide for Life Cycle Assessment - Detailed Guidance*, **2010**.

[37] F. Ilhan, D. S. Tyson, M. A. Meador, *Org. Lett.* **2006**, *8*, 577.

[38] T. Yamamoto, S. Wakabayashi, K. Osakada, *J. Organomet. Chem.* **1992**, *428*, 223.

[39] G. Majeau-Bettez, T. R. Hawkins, A. H. Strømman, *Environ. Sci. Technol.* **2011**, *45*, 4548.

[40] N. Greenwood, A. Earshaw, *Chemistry of the Elements*, **1984**.

[41] Y.-L. Xiao, X. Zhang, *In Encyclopedia of Reagents for Organic Synthesis - Nickel(II) Nitrate Hexahydrate*, **2017**.

[42] H. Siegel, M. Eggersdorfer, *Ullmanns's Encyclopedia of Industrial Chemistry*, **2002**.

[43] R. Miller, C. Abaecherli, A. Said, B. Jackson, *Ullmann's Encyclopedia of Industrial Chemstry*, **2012**.

[44] J. W. Wielandt, D. Ruckerbauer, *Inorg. Synth.* **2010**, *35*, 109.

[45] "https://environmentalgenome.org/wp-content/uploads/2017/09/ES_V4_triethylaluminum_2016-11-10_15-48.pdf," (last access on 12.02.2025)

[46] H. Lee, M. G. Campbell, R. Hernández Sánchez, J. Börgel, J. Raynaud, S. E. Parker, T. Ritter, *Organometallics* **2016**, *35*, 2923.

[47] P. B. Mackenzie, D. J. Krysan, *J. Org. Chem.* **1990**, *55*, 4229.

[48] "https://www.chemicalbook.com/ChemicalProductProperty_EN_CB2669337.htm.," (last access on 12.02.2025).

[49] D. E. C. Corbridge, *J. Am. Chem. Soc* **1996**, *33*, 7871.

[50] J. Cooke, *J. Chem. Educ.* **2019**, *96*, 2009.

[51] H. Schönhagen, R. Schmitz, *Pat. No. DE3513981A1* **1985**, 1.

[52] W. Hagui, K. Periasamy, J. F. Soulé, *European J. Org. Chem.* **2021**, 5388.

[53] P. A. Lyday, T. Kaiho, *Ullmann's Encycl. Ind. Chem.* **2015**, 1.
